# Three-dimensional analysis of anterior talofibular ligament strain patterns during cadaveric ankle motion using a miniaturized ligament performance probe

**DOI:** 10.1186/s12891-021-04058-2

**Published:** 2021-02-20

**Authors:** Yoshitaka Takeuchi, Ryota Inokuchi, Masato Takao, Mark Glazebrook, Xavier Martin Oliva, Takayuki Yamazaki, Maya Kubo, Danielle Lowe, Kentaro Matsui, Mai Katakura, Satoru Ozeki, Ankle Instability Group, Jorge Acevedo, Thomas Bauer, James Calder, Nuno Corte-Real, Mark Glazebrook, Stéphane Guillo, Jon Karlsson, John G. Kennedy, Gino M.M.J. Kerkhoffs, Siu Wah Kong, Peter G. Mangone, Frederick Michels, Andy Molloy, Caio Nery, Christopher Pearce, Anthony Perera, Hélder Pereira, Bas Pinenburg, Fernando Raduan, James W. Stone, Masato Takao, Yves Tourné, Jordi Vega, Jin Woo Lee

**Affiliations:** 1 Department of Orthopaedic Surgery, Dokkyo Medical University Saitama Medical Center, 2-1-50, Minamikoshigaya, Koshigaya, Saitama, Japan; 2 Clinical and Research Institute for Foot and Ankle Surgery, 341-1, Mangoku, Kisarazu, 292-0003 Chiba, Japan; 3 Department of Health Services Research Faculty of Medicine, University of Tsukuba, 1-1-1 Tenno-dai, Ibaraki Tsukuba, Japan; 4 Division of Orthopaedic Surgery, Dalhousie University and the Queen Elizabeth to health Sciences center, 1796 Summer St, Nova Scotia Halifax, Canada; 5 Department of Human Anatomy, University of Barcelona, Calle Casanova, 143, 08038 Barcelona, Spain; 6 Department of Orthopaedic Surgery, Teikyo University, 2-11-1 Kaga, Itabashi, Tokyo, Japan; 7 Division of Orthopaedic Surgery, Lions Gate Hospital, 231 East 15th Street, BC North Vancouver, Canada

**Keywords:** Strain pattern, ankle, Strain gauge, Anterior talofibular ligament, MLPP, ATFL

## Abstract

**Background:**

Measuring the strain patterns of ligaments at various joint positions informs our understanding of their function. However, few studies have examined the biomechanical properties of ankle ligaments; further, the tensile properties of each ligament, during motion, have not been described. This limitation exists because current biomechanical sensors are too big to insert within the ankle. The present study aimed to validate a novel miniaturized ligament performance probe (MLPP) system for measuring the strain patterns of the anterior talofibular ligament (ATFL) during ankle motion.

**Methods:**

Six fresh-frozen, through-the-knee, lower extremity, cadaveric specimens were used to conduct this study. An MLPP system, comprising a commercially available strain gauge (force probe), amplifier unit, display unit, and logger, was sutured into the midsubstance of the ATFL fibers. To measure tensile forces, a round, metal disk (a “clock”, 150 mm in diameter) was affixed to the plantar aspect of each foot. With a 1.2-Nm load applied to the ankle and subtalar joint complex, the ankle was manually moved from 15° dorsiflexion to 30° plantar flexion. The clock was rotated in 30° increments to measure the ATFL strain detected at each endpoint by the miniature force probe. Individual strain data were aligned with the neutral (0) position value; the maximum value was 100.

**Results:**

Throughout the motion required to shift from 15° dorsiflexion to 30° plantar flexion, the ATFL tensed near 20° (plantar flexion), and the strain increased as the plantar flexion angle increased. The ATFL was maximally tensioned at the 2 and 3 o’clock (inversion) positions (96.0 ± 5.8 and 96.3 ± 5.7) and declined sharply towards the 7 o’clock position (12.4 ± 16.8). Within the elastic range of the ATFL (the range within which it can return to its original shape and length), the tensile force was proportional to the strain, in all specimens.

**Conclusion:**

The MLPP system is capable of measuring ATFL strain patterns; thus, this system may be used to effectively determine the relationship between limb position and ATFL ankle ligament strain patterns.

## Background

Measuring the strain patterns of joint ligaments, in several different joint positions, is necessary to understand their function. For example, the biomechanical properties of large joints have been defined by directly measuring the tensile strength of their associated ligaments [1–4]. However, very few studies have explored the biomechanical properties of the ankle [5, 6] or the tensile properties of each ligament during ankle motion. This is because the existing biomechanical sensors, used in large joint research, are too big to fit within the ankle ligaments.

We previously used a custom-made ankle ligament testing device to directly measure the load on each ankle ligament [6]. The device used a small force probe in a custom-made ankle ligament and clarified the tensile pattern of the anterior talofibular, calcaneofibular, posterior talofibular, and tibiocalcaneal ligaments during passive circumferential rotating motions of the ankle and subtalar joints. However, because the sensors were custom-made, they only have limited usefulness under other conditions.

In this study, we developed a novel, miniaturized ligament performance probe (MLPP) system that can be inserted into small ligaments to facilitate the measurement of their biomechanical properties. We aimed to validate MLPP anterior talofibular ligament (ATFL) strain pattern measurements during ankle motion.

## Methods

### Components of the MLPP system

**Fig. 1:**
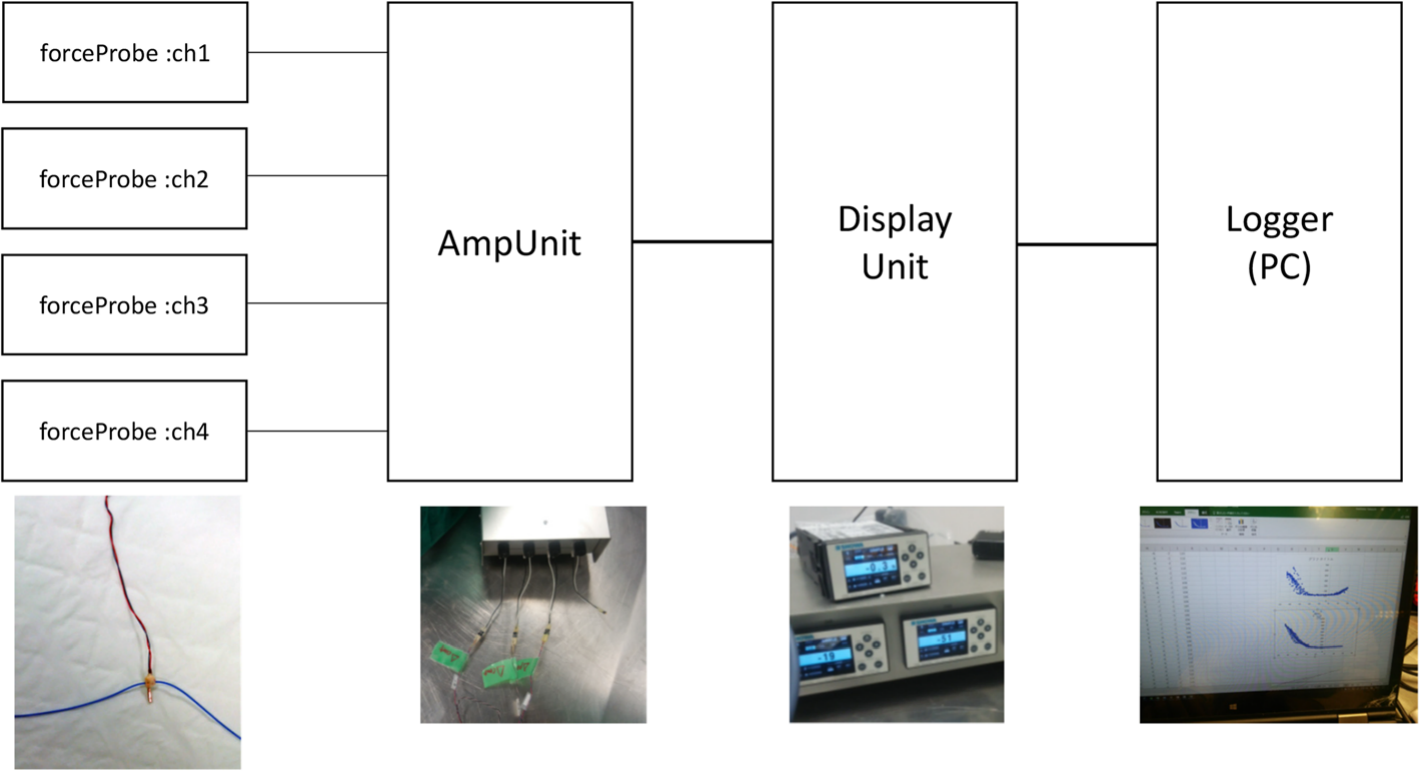
Miniaturized ligament performance probe system. The system is composed of a force probe (left), an amplifier unit (middle left), a display unit (middle right), and a logger (right)

The MLPP system is composed of a commercially available strain gauge (force probe), amplifier unit, display unit, and logger (Fig. 1), allowing the system to detect and report small changes in resistance. These resistance changes are enlarged by the bridge of the amplifier unit and transferred to the display unit output, where analog-to-digital conversion takes place and the degree of strain is displayed. This strain measurement is also converted to an analog value, and its voltage is recorded in the logger.

**Fig. 2:**
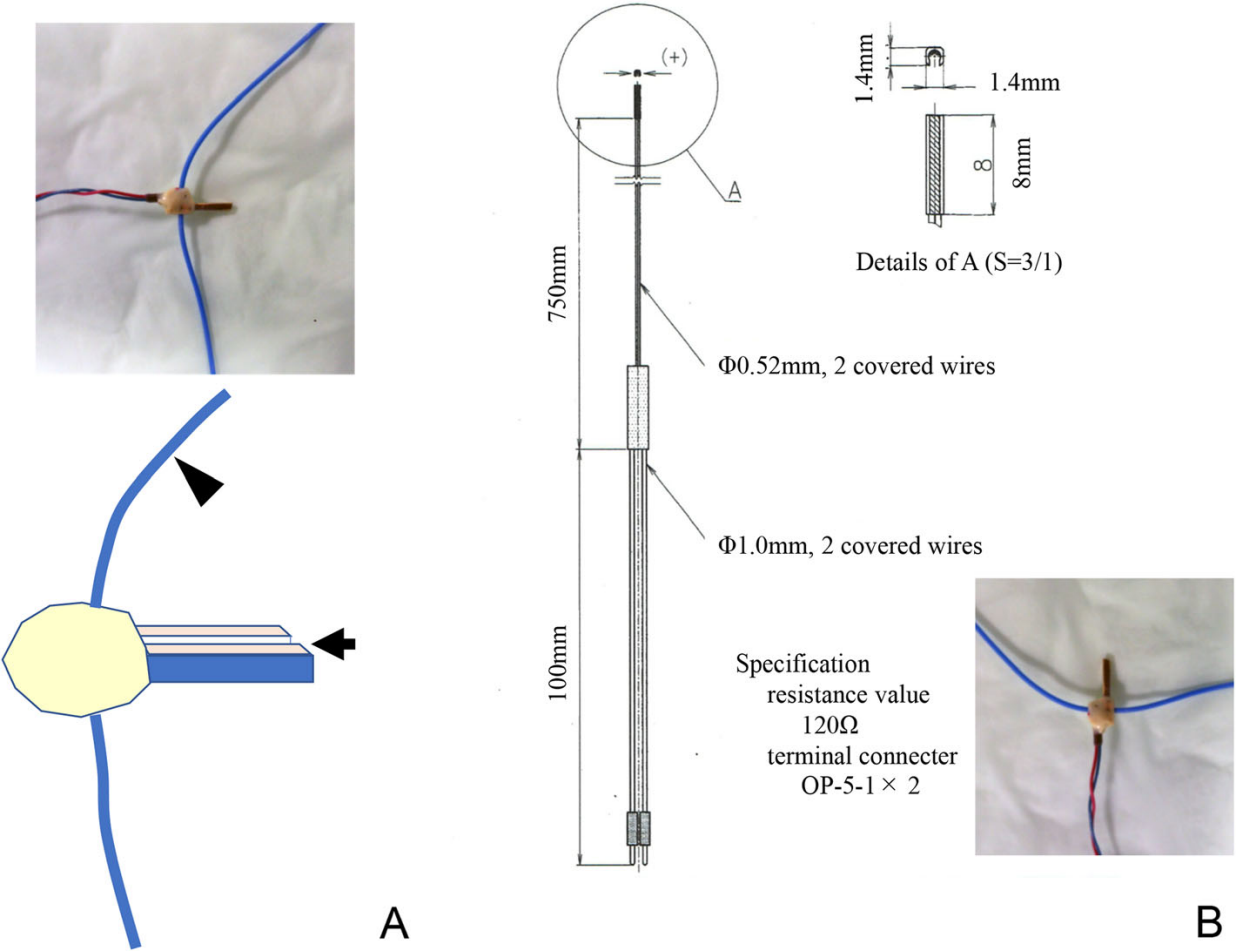
Force probe (strain gauge). A rectangular force probe (**a**) with a tubular structure that has slits extending vertically along one side of its surface (**b**) is shown

**Fig. 3:**
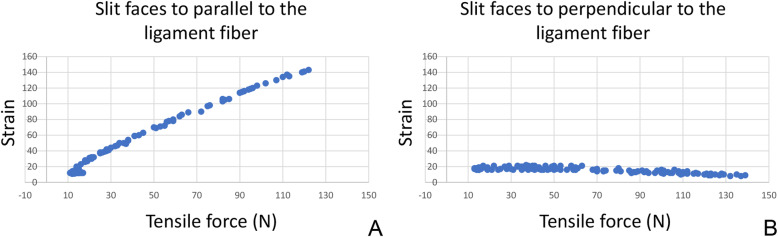
Measured value differences that occur by altering the force probe slit angle. The ligament tensile pattern can be measured if the slit of the force probe is parallel to the ligament fiber, as shown in (**a**). However, the values are skewed if the slit on the force probe is perpendicular to the ligament fiber, as shown in (**b**)

**Fig. 4:**
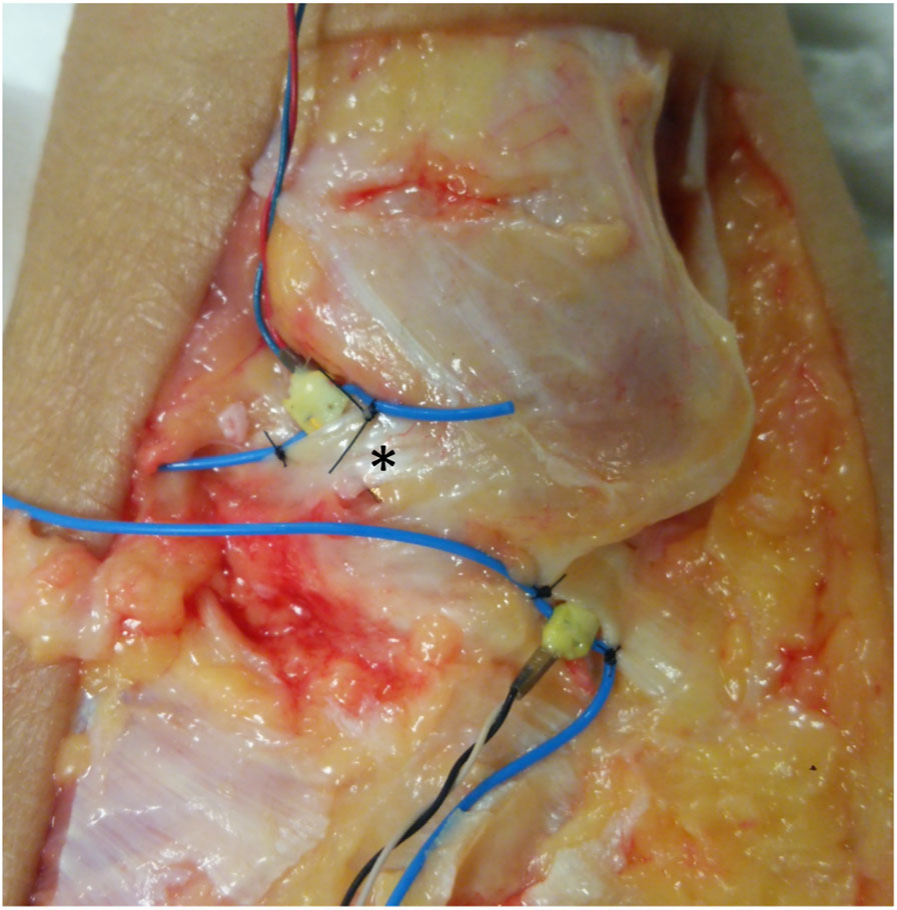
Insertion and fixation of the force probe into the anterior talofibular ligament. Both ends of the tube are sutured to the anterior talofibular ligament (*) to prevent rotation of the force probe within the ligament

The force probe (Showa Unilateral Strain Gauge; Showa Measuring Instruments, Tokyo, Japan) is rectangular (width, 2 mm; height, 1.5 mm; length, 8 mm) and has a tubular structure, with slits that extend vertically along one side of its surface (Fig. 2a and b). In the force probe, the internal strain gauge is distorted by applying force in a certain direction, allowing the strain to be measured. When the force probe is inserted into tissue, it may rotate as forces are applied, which may reduce or invert the output (Fig. 3a and b). To avoid this rotational influence, a tube for preventing rotation was attached to the force probe, and both ends were sutured to the target tissue (Fig. 4).

**Fig. 5:**
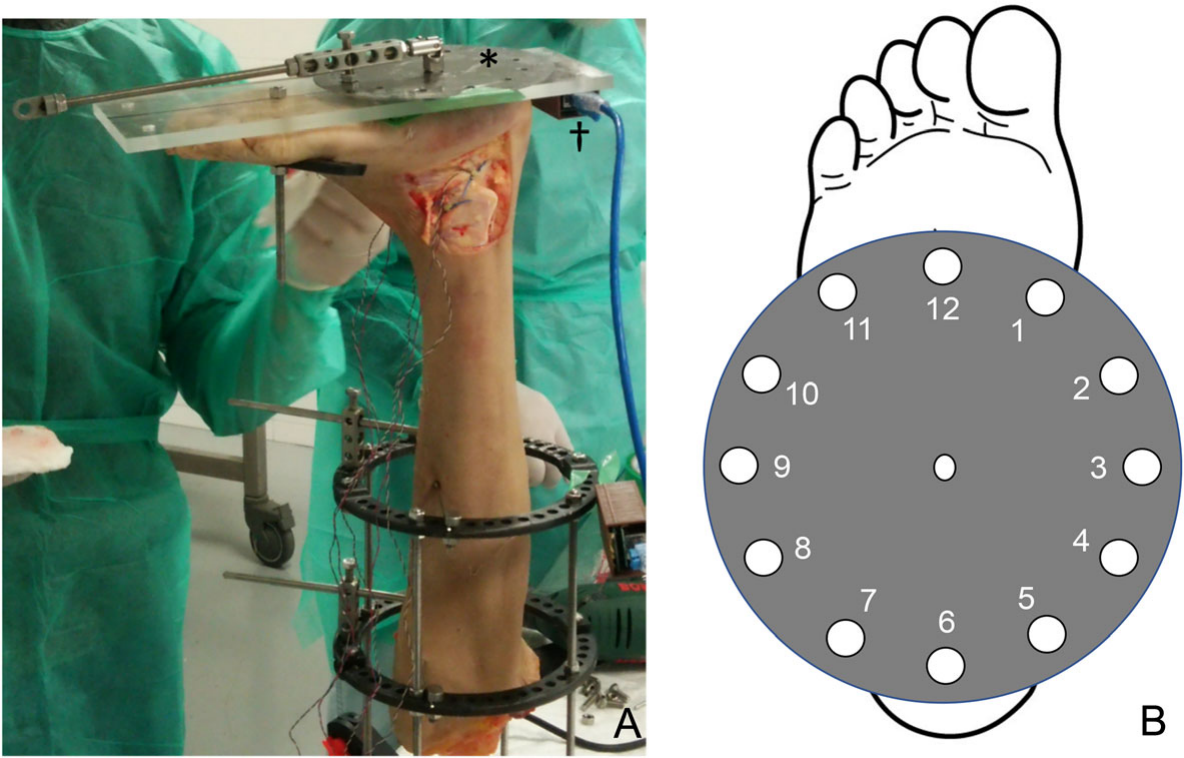
Specimen positioning. A cadaveric lower limb is shown vertically positioned using an Ilizarov ring-shaped external fixator (**a**). A clock (*, **b**) and performance cube (†) are shown affixed to anacrylic plate

A performance cube was used to measure the position of the ankle (Fig. 5). The cube is composed of a nine-axis sensor (MPU-9250; TDK InvenSense, San Jose, CA, USA), microcontroller (ESP32), and logger. The MPU-9250 and ESP32 were loaded into the performance cube. The MPU-9250 is a sensor that records positional information and measures the values of nine axes, in total; it also records angular acceleration and geomagnetism. The MPU-9250 is equipped with a digital motion processor that automatically measures motion at the time of sensor initialization and determines posture. The ESP32 is a microcontroller that records data obtained from MPU-9250 and transmits it to the logger using a wireless module. The performance cube was synchronized with the MLPP system.

### Cadavers

Six fresh-frozen, through-the-knee, lower extremity cadaveric specimens were used for this study (three right and three left legs). Three specimens were from male and three were from female cadavers. The median age of each cadaver, at the time of death, was 64 years (range, 46–82 years). The specimens were free of ankle or hindfoot deformities, had not undergone surgery or dissection, and did not have histories of trauma or other anatomy-altering pathologies. All cadaveric studies were performed at the University of Barcelona (Catalonia, Spain), and all methods used in this study were reviewed and approved by the university’s institutional review board. Consent for the storage and use of the bodies for research purposes was given by the donors prior to their deaths or by their next of kin.

### Investigating AFTL strain patterns

The procedures described in this section were performed by an experienced foot and ankle surgeon. An incision was made in the lateral ankle of each specimen, and the ATFL was exposed. A force probe, in a force probe tube, was placed into the midsubstance of the ATFL, and the slit in the force probe was aligned with the long axis of the ligament fiber. After placing the force probe into the ligament, the force probe tube was sutured to the ligament fibers using 3 − 0 nylon sutures to prevent force probe rotation (Fig. 4).

An Ilizarov ring-shaped external fixator was placed on the lower leg, and the lower limb was fixed vertically, relative to the measurement desk, using a vice to allow localization of the distal upper and proximal lower portions of each specimen. A round metal disk (a “clock”, diameter 150 mm), with 6-mm-diameter holes placed every 30° around its circumference, was affixed to an acrylic plate (width, 120 mm; length, 280 mm; thickness, 10 mm). The plate was fixed to the plantar aspect of the foot with a 6-mm-diameter screw inserted into the calcaneus; an 8-mm-diameter rod was also inserted between the second and third metatarsals (Fig. 5a). The plate had a 25-cm arm, and a 0.5-kg weight was added to its end to approximate the application of a 1.2-Nm force (0.5 kg × 0.25 m × 9.80665 = 1.23 N m) to the ankle and subtalar joint complex. The arm of the plate was rotated to each position (in 30° increments) on the clock, allowing for the measurement of AFTL strain at each of its ends (Fig. 5b). The ankle positions corresponding to dorsiflexion, plantar flexion, inversion, and eversion were achieved when the plate arm was at the 12-, 6-, 3-, and 9-o’clock positions, respectively. The axial motion angles for dorsiflexion and plantar flexion were measured using the electronic goniometer (MPU-9250), which was synchronized with the MLPP system. After all measurements in the intact specimens were completed, the ATFL was cut at the fibular attachment points to free the force probe.

### Data analysis

The relationships between the foot positions and the AFTL tensile forces were analyzed. The tensile force data from the force probe were obtained by synchronizing the probe with the clock’s arm at each 30° stop. The ankle was moved from 15° dorsiflexion to 30° plantar flexion 10 times, manually, and the strain on the ATFL during the ankle motion was measured. Individual strain data points were aligned with the values at the neutral (0) position; the maximum value was 100. The values at each position were connected with a line, and the ligament tension patterns of the specimens were compared. All strain data are presented as means ± standard deviations (SD).

## Results

**Fig. 6:**
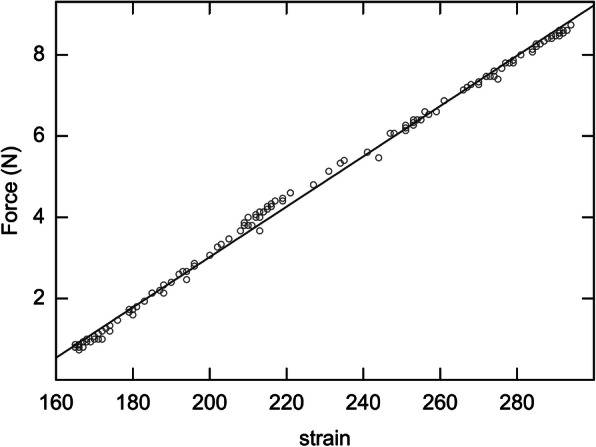
Tensile force data. The tensile force data collected from the anterior talofibular ligament during ankle motion from full dorsiflexion to full plantar flexion (10 times), are shown

**Fig. 7:**
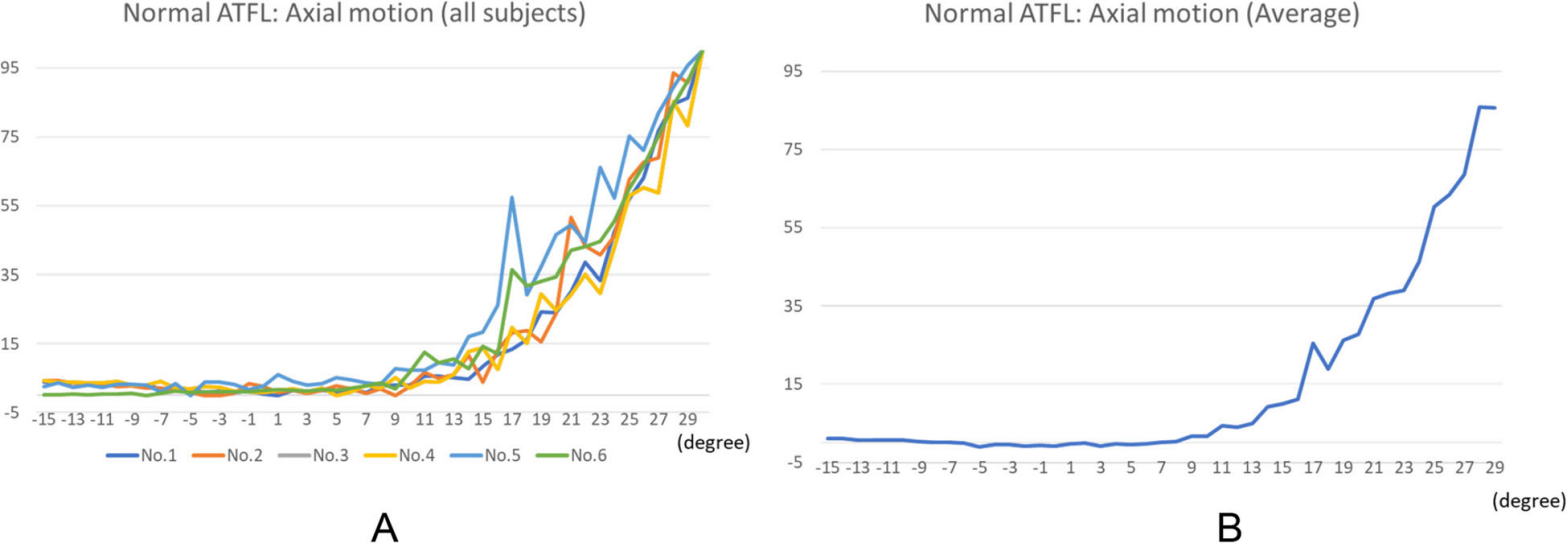
Strain patterns of the anterior talofibular ligaments. The strain patterns, at each endpoint, acquired during ankle motion from 15° dorsiflexion to 30° plantar flexion are shown. Both the individual values (**a**) determined for each of the six specimens and the average values (**b**) are shown

**Fig. 8:**
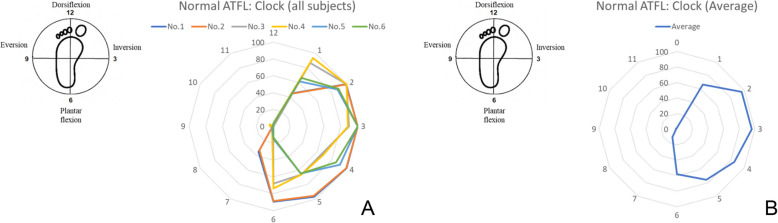
Tensile pattern at each end of the anterior talofibular ligament on the clock. The tensile patterns for each of the individual specimens (**a**) and the average values (**b**) are shown

Within the elastic range of the ATFL, the tensile force was determined to be proportional to the strain, in all cases (Fig. 6). The strain patterns, at each endpoint, for the ATFLs of specimens that underwent the test motions (from 15° dorsiflexion to 30° plantar flexion) are shown in Fig. 7 and on the clock diagram (Fig. 8). Throughout this motion, the ATFL tensed at approximately 20° (plantar flexion) and the tensile strength increased as the plantar flexion angle increased. The ATFL was maximally tensioned at the 2- and 3-o’clock (inversion) positions (96.0 ± 5.8 and 96.3 ± 5.7, respectively) and the tension declined sharply towards the 7-o’clock position (12.4 ± 16.8). All specimens showed similar strain patterns, with axial and clock motion coefficients of variation of 0–1 and 0.05–1, respectively.

## Discussion

Our results showed that the MLPP system provided results that were similar to those determined using our previous system. The sensor used in the MLPP system is commercially available and widely used; its accuracy is guaranteed. In addition, the insertion technique is simple, facilitating the measurement of the ATFL strain pattern throughout the ankle motion.

Previous studies showed that using Roentgen measurements [7], an Inman ankle machine [8], a magnetic position and orientation trading system [9], a video-based data collection system [10], and the 3SPACE® FASTRAK® system [11, 12], allow the direct measurement of the tension pattern for each ligament in the ankle. However, these systems only allow indirect estimations of the ankle ligament biomechanical properties. For the direct measurement of ligament load, the sensor needs to be inserted into the ligament. DeRouin et al. [13] reported the use of a system that employed a wireless sensor for measuring ligament tensile force in large joints. It had a high-force sensor that consisted of a stainless-steel strip with hooks at both ends. Although the system was able to directly measure the load applied to the ligament, the sizes of the metal strip (28 × 1 × 0.5 mm) and sensor (20 × 1 × 30 µm) were too large to allow their use in small joints, such as the ankle.

We previously developed an ankle ligament testing device that could directly measure each ligament’s tensile pattern and showed the ATFL was maximally tensioned at the 2- and 3-o’clock (inversion) positions [6]; the devices primary downfall was that it was custom-made and, therefore, not readily available. The MLPP system, described in the present study, is made of commercially available products that can be inserted into small ligaments, facilitating the precise measurement of AFTL strain patterns during ankle motion. In addition, we previously confirmed the tension pattern of the deltoid ligament using this system [14]. Thus, the MLPP system may be used to improve surgical repair precision and improve post-injury ATFL reconstructions, in the future.

### Limitations

A disadvantage of the MLPP system is that it measures the strain value of the ligament instead of the tensile force. Within the elastic range of the ATFL (the range within which the ligament can return to its original shape and length), force and strain were linearly proportional. Therefore, the strain value can theoretically be converted to newton-force, if Young’s modulus is obtained via calibration. However, accurately determining Young’s modulus is difficult because the water content of the tissue decreases over time and the ligament elasticity changes. Thus, the small degree of variation observed in this study may have been influenced by temporal changes in ligament elasticity.

## Conclusions

This study described a new MLPP system that is able to effectively establish the relationship between the limb position and ATFL strain pattern. This ability will enable an enhanced understanding of the biomechanical function of the ATFL.

## Data Availability

The data sets used and/or analyzed during the current study are available from the corresponding author on reasonable request.

## Abbreviations

ATFL: Anterior talofibular ligament
MLPP: Miniaturized ligament performance probe

## References

[1] Ahmed AM, Hyder A, Burke DL, Chan KH (1987). In-vitro ligament tension pattern in the flexed knee in passive loading. J Orthop Res.

[2] Ahmed AM, Burke DL, Duncan NA, Chan KH (1992). Ligament tension pattern in the flexed knee in combined passive anterior translation and axial rotation. J Orthop Res.

[3] Markolf KL, Willems MJ, Jackson SR, Finerman GA (1998). In situ calibration of miniature sensors implanted into the anterior cruciate ligament part II: force probe measurements. J Orthop Res.

[4] Bahr R, Pena F, Shine J, Lew WD, Engebretsen L (1998). Ligament force and joint motion in the intact ankle: a cadaveric study. Knee Surg Sports Traumatol Arthrosc.

[5] Colville MR, Marder RA, Boyle JJ, Zarins B (1990). Strain measurement in lateral ankle ligaments. Am J Sports Med.

[6] Ozeki S, Kitaoka H, Uchiyama E, Luo ZP, Kaufman K, An KN (2006). Ankle ligament tensile forces at the end points of passive circumferential rotating motion of the ankle and subtalar joint complex. Foot Ankle Int.

[7] Weseley MS, Koval R, Kleiger B (1969). Roentgen measurement of ankle flxion-extension motion. Clin Orthop Relat Res.

[8] Fumich RM, Ellison AE, Guerin GJ, Grace PD (1981). The measured effect of taping on combined foot and ankle motion before and after exercise. Am J Sports Med.

[9] An KN, Jacobsen MC, Berglund LJ, Chao EY (1988). Application of a magnetic tracking device to kinesiologic studies. J Biomech.

[10] Kepple TM, Stanhope SJ, Lohmann KN, Roman NL (1990). A video-based technique for measuring ankle-subtalar motion during stance. J Biomed Eng.

[11] Kitaoka HB, Luo ZP, An KN (1997). Three-dimensional analysis of normal ankle and foot mobility. Am J Sports Med.

[12] Knudson GA, Kitaoka HB, Lu CL, Luo ZP, An KN (1997). Subtalar joint stability. Talocalcaneal interosseous ligament function studied in cadaver specimens. Acta Orthop Scand.

[13] DeRouin A, Pacella N, Zhao C, Ong KG (2016). A wireless sensor for real-time monitoring of tensile force on sutured wound sites. IEEE Trans Biomed Eng.

[14] Takao M, Ozeki S, Oliva XM, Inokuchi R, Yamazaki T, Takeuchi Y, Kubo M, Lowe D, Matsui K, Katakura M (2020). Ankle Instability Group, Glazebrook M. Strain pattern of each ligamentous band of the superficial deltoid ligament: a cadaver study. BMC Musculoskelet Disord.

